# A bibliometric analysis of 16,826 triple-negative breast cancer publications using multiple machine learning algorithms: Progress in the past 17 years

**DOI:** 10.3389/fmed.2023.999312

**Published:** 2023-02-08

**Authors:** Kangtao Wang, Chanjuan Zheng, Lian Xue, Dexin Deng, Liang Zeng, Ming Li, Xiyun Deng

**Affiliations:** ^1^Department of General Surgery, The Xiangya Hospital, Central South University, Changsha, Hunan, China; ^2^Key Laboratory of Model Animals and Stem Cell Biology in Hunan, Department of Pathophysiology, School of Medicine, Hunan Normal University, Changsha, Hunan, China; ^3^Xiangya School of Medicine, Central South University, Changsha, Hunan, China; ^4^Department of Pathology, Guangzhou Women and Children’s Medical Center, Guangdong Provincial Clinical Research Center for Child Health, Guangzhou, China; ^5^Department of Immunology, College of Basic Medical Sciences, Central South University, Changsha, Hunan, China

**Keywords:** machine learning, bibliometric analysis, Latent Dirichlet Allocation, triple-negative breast cancer, Nanoparticle research

## Abstract

**Background:**

Triple-negative breast cancer (TNBC) is proposed at the beginning of this century, which is still the most challenging breast cancer subtype due to its aggressive behavior, including early relapse, metastatic spread, and poor survival. This study uses machine learning methods to explore the current research status and deficiencies from a macro perspective on TNBC publications.

**Methods:**

PubMed publications under “triple-negative breast cancer” were searched and downloaded between January 2005 and 2022. R and Python extracted MeSH terms, geographic information, and other abstracts from metadata. The Latent Dirichlet Allocation (LDA) algorithm was applied to identify specific research topics. The Louvain algorithm established a topic network, identifying the topic’s relationship.

**Results:**

A total of 16,826 publications were identified, with an average annual growth rate of 74.7%. Ninety-eight countries and regions in the world participated in TNBC research. Molecular pathogenesis and medication are most studied in TNBC research. The publications mainly focused on three aspects: Therapeutic target research, Prognostic research, and Mechanism research. The algorithm and citation suggested that TNBC research is based on technology that advances TNBC subtyping, new drug development, and clinical trials.

**Conclusion:**

This study quantitatively analyzes the current status of TNBC research from a macro perspective and will aid in redirecting basic and clinical research toward a better outcome for TNBC. Therapeutic target research and Nanoparticle research are the present research focus. There may be a lack of research on TNBC from a patient perspective, health economics, and end-of-life care perspectives. The research direction of TNBC may require the intervention of new technologies.

## Highlights

- All Triple-negative breast cancer (TNBC) publications in the PubMed database from 2005 to 2021 were included in the analysis.
- Triple-negative breast cancer research mainly focused on three aspects: Therapeutic target research, Prognostic research, and Mechanism research.
- Therapeutic target research and Nanoparticle research are the present research focus.
- The Latent Dirichlet Allocation (LDA) algorithm we built is a convenient tool that can help researchers discover changes in research focus from medical text big data.

## 1. Background

Breast cancer currently accounts for 30% of newly diagnosed malignant tumors in women and causes 15% of women to die from cancer (1). For the first time, Perou described the intrinsic molecular subtypes of breast cancer and described Triple-negative breast cancer (TNBC) in 2000 using complementary DNA microarray technology (2). Furthermore, TNBC is the most aggressive subtype of breast cancer, accounting for about 10–20% of breast cancer cases (3, 4). TNBC is still unsatisfactory in diagnosis and treatment.

Bibliometrics is a quantitative analysis method of academic publications, which can discover the progress of discipline research from a macro perspective and provide support for future research directions (5). TNBC-related literature information analysis is scarce. Teles et al. (6) conducted a bibliometric study of 1,932 publications in 2018 to study nanomedicine research’s global trend on TNBC. However, the inclusion criteria of this study are too broad, and the analysis methods are insufficient to analyze the *status quo* of the TNBC study. Unfortunately, bibliometric studies on TNBC remain insufficient due to the lack of practical language analysis tools to integrate metatext data.

Natural Language Processing (NLP) is a computing technology used to analyze human language, a part of machine learning (7). Various algorithms have been successfully applied to deal with medical information (8). Latent Dirichlet Allocation (LDA) is bibliometrics’s most classical topic modeling method to present many unstructured texts and information (9, 10). LDA can perform topic analysis on texts (5). We recently constructed LDA and NLP methods to analyze more than 23,000 rectal cancer-related publications between 1994 and 2018. We have found the research deficiencies in the last 25 years and predicted the future research focus (11). Therefore, through the use of mature LDA methods and machine learning techniques to discover the current research from a macro perspective, at the same time discover the missing research topics in the past, and predict potential research breakthroughs in the future.

We analyzed all past TNBC publications indexed by PubMed under Triple-negative breast cancer in the present study. We improved our algorithm based on previous research and conducted a more detailed analysis of all TNBC publications with more visual expression to highlight current research focus in TNBC, research deficiencies, and specific areas with future opportunities.

## 2. Materials and methods

### 2.1. Research design

**FIGURE 1:**
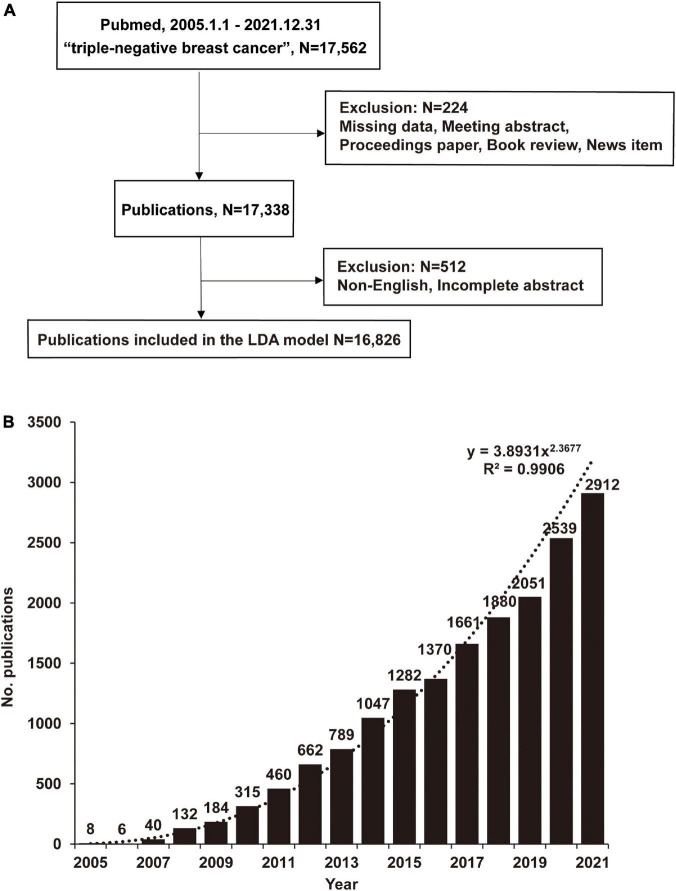
The number of publications on triple-negative breast cancer (TNBC) has increased rapidly in recent 17 years. **(A)** Using the search terms “triple-negative breast cancer” in the PubMed database, download publications through the R pubquery package. Missing data or when the publication was a meeting abstract, proceedings paper, a correction, a book review, or a news item were manually excluded, and finally, 17,338 publications were included in the general analysis. Latent Dirichlet Allocation (LDA) analyzed 16,826 publications. **(B)** Publications analyzed by LDA, Python. Data were visualized using Excel. The number of publications is shown yearly, and y = 3.8931x^2.3677^ (R^2^ = 0.9906) is the fitted function.

The study design was based on the basic rules of bibliometrics, as shown in Figure 1 for a flowchart (12, 13). The study used a two-stage structured approach to bibliometric analysis and visual assessment of published scientific literature. Provide an understanding based on the data and the researcher’s professional background. The PubMed database^1^ is a biomedical specialty database that provides multiple search strategies and is a free, publicly available database. For this research, the PubMed database, which contains an application programming interface (API) that can export abstracts, was used, and publications containing abstracts were downloaded for analysis.

### 2.2. Inclusive and exclusive criteria

Table 1 shows the steps to obtain full TNBC-related publications in the PubMed database. All publications under Triple Negative Breast Cancer were downloaded between January 1, 2005, and January 1, 2022. There are 17,562 publications. Missing data, conference abstracts, conference proceedings, book reviews, and news items were excluded, and 17,338 publications were ultimately included in the bibliometric analysis (Figure 1A). Details of inclusion and exclusion are shown in Table 2. After excluding non-English publications and incomplete abstracts, the final 16,826 publications were analyzed by the LDA algorithm to obtain the focus changes and their relevance of research topics in publications in this field. The whole record of search results is downloaded in XML format *via* R’s easyPubMed package. Data extracted from R^2^ and Python^3^, including publication year, abstract, study types, geographic information, and Medical Subject Headings (MeSH) terms, were obtained.

**TABLE 1:** Triple-negative breast cancer (TNBC) publications assortment steps.

Exploration steps	Query on PubMed	Description
1	Triple negative breast cancer	(“triple negative breast neoplasms”[MeSH Terms] OR (“triple”[All Fields] AND “negative”[All Fields] AND “breast”[All Fields] AND “neoplasms”[All Fields]) OR “triple negative breast neoplasms”[All Fields])
2	Data duration	(2005:2021[pdat])

**TABLE 2:** Inclusive and exclusive criteria.

Parameter of selection of a publication	Inclusion criterion	Exclusion criterion	Rationale for inclusion–exclusion
Language	English	Other languages	The working language of the LDA algorithm is English. Other languages are not recognized
Publication date	2005–2021	Publications before 2005 and after 2021	Not included in the 2022 publication as it has not been fully published
Publication type	All	Missing data, meeting abstract, proceeding paper, book review, news item	As the LDA algorithm is unsupervised machine learning, the analysis must include abstract as the text editor. In addition to incomplete content, try to include research articles and reviews.
Funding sponsor	All	No exclusion	This parameter does not affect the selection criterion
Affiliation/organization	All	No exclusion	This parameter does not affect the selection criterion
Funding	All	No exclusion	This parameter does not affect the selection criterion
Country	All	No exclusion	Publication from each country has its significance

### 2.3. LDA and algorithms and analytical methods

Latent Dirichlet Allocation was used to identify more specific research topics in each article. Python was used to model the topics by analyzing the abstracts of all indexed articles in the record. Topics were set at 50. The criteria for selecting the number of topics were perplexity, redundancy, and legibility. Based on the algorithmic calculation of topic probability, we finally determined the topic to which each article belongs. Next, we manually checked the names of each glossary based on the abstract. Finally, we used the Louvain algorithm and Gephi to perform cluster analysis to establish a topic network to determine the relationship between topics (14). We identified the two topics with the highest attribution probability in each publication, counted the number of simultaneous occurrences in each document, and established links between topics.

All the original data were uploaded and publicly available, including all retrieval methods, algorithm codes, and raw literature data in this article (Figure 1A). The literature search and download code can be obtained on R by easyPubMed package^4^. The R code is publicly available on GitHub^5^. We have uploaded relevant Python code on GitHub^6^, Zenodo^7^ and LDA code (Supplementary LDA coding-updated). The network visualization in this article is carried out using the software package Gephi^8^. This study used publicly published data and did not need approval by the relevant institutional review board or ethics committee. A step-by-step instruction is provided in the Supplementary material to facilitate the reader to understand further the research details (Supplementary information 1).

## 3. Results

### 3.1. The number of publications in TNBC research increases every year

We identified and analyzed 16,826 publications from January 2005 to 2022 (Figure 1B). The annual growth trend aligns with the fitting curve y = 3.8931x^2.3677^ (R^2^ = 0.9906). An average of 1,019 publications are published each year, with an average annual growth rate of 74.7%. It is expected that 3,650 publications will be published in 2022. Among all publications, 1,646 journals have publications on TNBC. We identified the ten most popular journals that published 3,118 publications, accounting for 18.0% of all publications (Supplementary Table 1). Therefore, emphasizing posts from these key journals helps us keep up with the latest trends. *Breast Cancer Research and Treatment*, *PLoS One*, and *Scientific Reports* are the top three journals with 690, 427, and 331 publications.

### 3.2. The proportion of clinical trials in TNBC publications has increased every year

**FIGURE 2:**
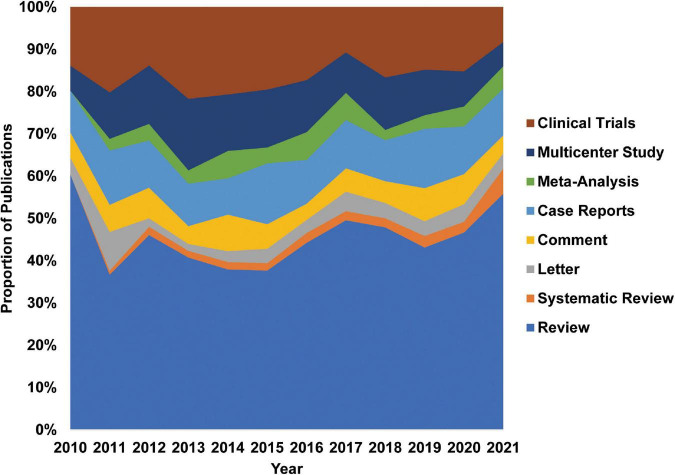
Clinical trials and multicenter studies have a large proportion of research. We divide publications into eight categories according to the types provided in the database. Data were shown by percentage.

To explore the research fields of TNBC, we first divided the publications into nine categories according to the fields provided by the database from 2010 in cancer research and set them as 100 per cent (Figure 2). We found that clinical trials and multicenter studies accounted for 25% of publications. The proportions of reviews and meta-analyses increased from 35% in 2011 to 50% in 2021. Since high-quality meta-analysis is generally considered a clinically guiding study, it is reasonable to expect that the publication of TNBC meta-analysis will increase. Many clinical trials of TNBC have been improved and will continue to improve its clinical practice.

### 3.3. The United States and China have the highest number of publications in the field of TNBC

**FIGURE 3:**
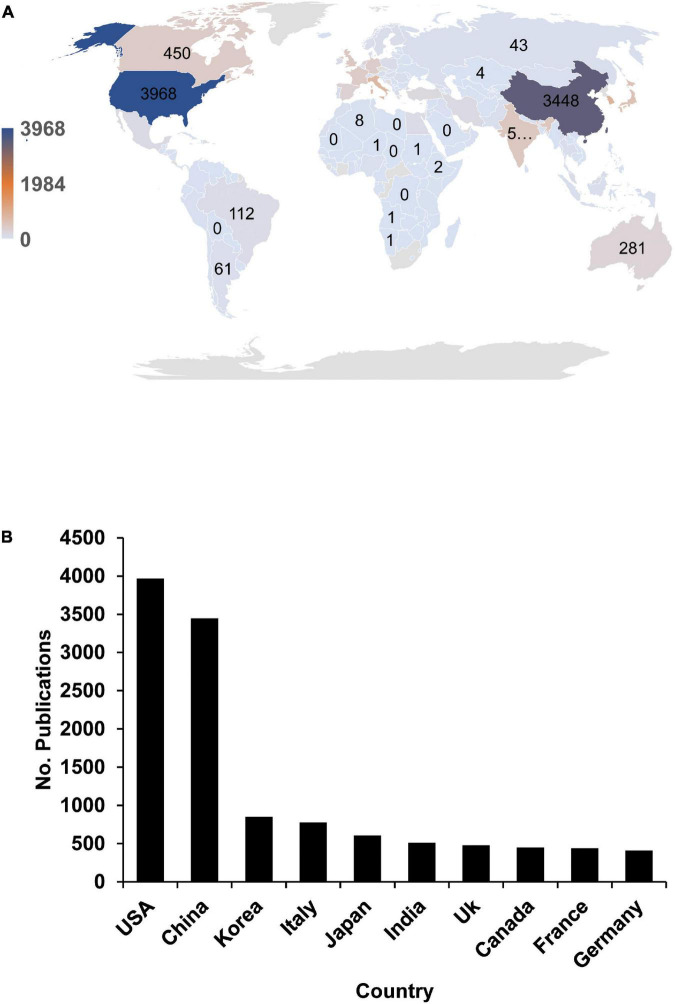
Global triple-negative breast cancer (TNBC) research differs significantly between regions. **(A)** The global distribution of TNBC publications in the recent 17 years is shown. We extracted the country information based on the first publication’s affiliation. **(B)** Top 10 countries with the highest publication numbers in TNBC research.

To further understand the global TNBC research situation, we analyzed the geographic information by research institutions. We found that 98 countries or regions worldwide have publications on TNBC (Figure 3A). The top 10 countries’ publications accounted for 78.2%, indicating a pronounced head effect. Moreover, more than half of the publications were derived from the United States, China, Korea, and Italy, accounting for 25.0%, 21.8%, 5.4%, and 4.9% of all publications, respectively (Figure 3B). This phenomenon reminds us that the vast majority of the global population has participated in TNBC research, especially in the northern hemisphere.

### 3.4. Molecular pathogenesis and medication are most studied in TNBC research

**FIGURE 4:**
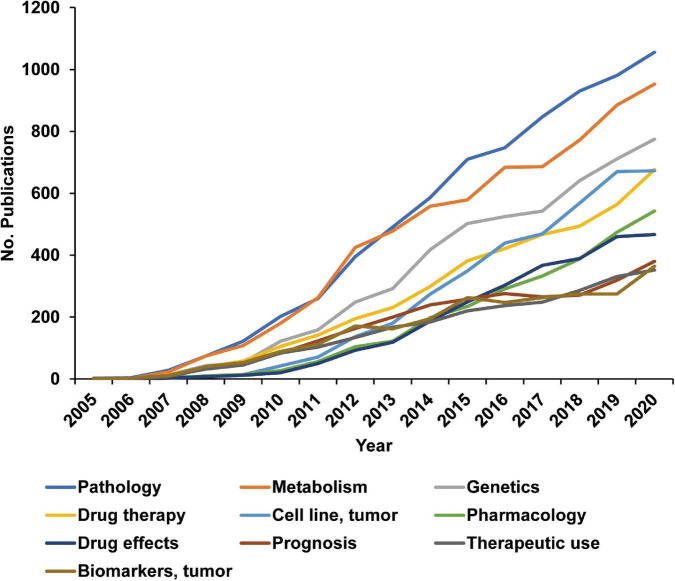
Molecular pathogenesis and medication are most studied in triple-negative breast cancer (TNBC) research. Each publication contains several Medical Subject Headings terms to describe the research content roughly. R was used to analyze the themes of the publications through Medical Subject Headings terms. The figure shows the most researched topics in the last 16 years.

MeSH terms can represent the research content of the publications. A total of 6,288 MeSH terms appeared 248,250 times in all 16,826 publications, indicating that the studies covered multiple aspects (Supplementary Table 2). The top 10 cited MeSH terms are listed in Figure 4. Both pathology and metabolism have appeared more than 7,000 times, suggesting that the research on TNBC focused on exploring its molecular pathogenesis. In addition, 5 of the top 10 cited MeSH terms are directly related to medication research. Therefore, we infer that pathogenic mechanism and medication research will continue to focus on TNBC research in the foreseeable future.

### 3.5. LDA results: TNBC research focus on therapeutic target research, prognostic research, and mechanism research

**FIGURE 5:**
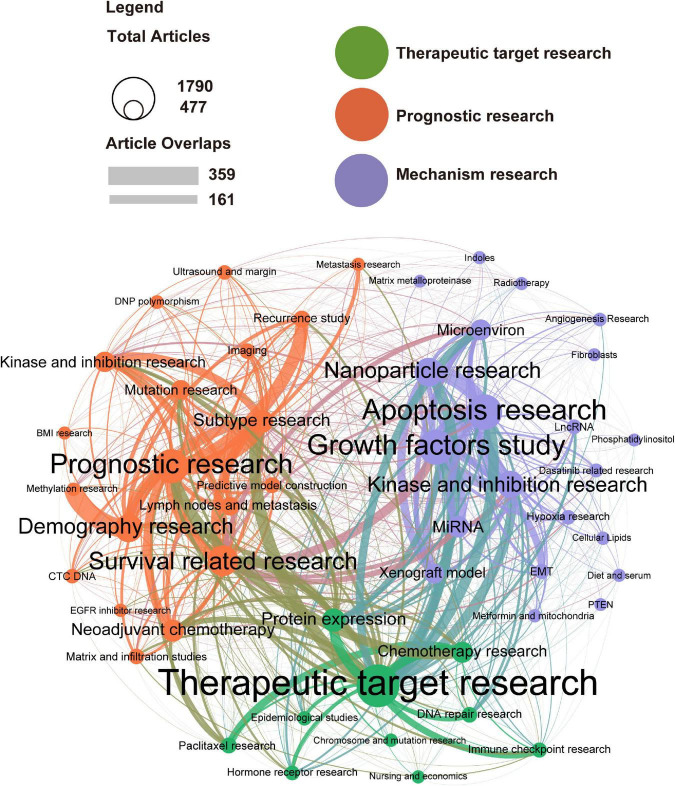
Latent Dirichlet Allocation (LDA) identified that the triple-negative breast cancer (TNBC) research is focused on three areas Therapeutic target research, Prognostic research, and Mechanism research. Topic cluster network studied by Latent Dirichlet Allocation: inter-and intra-relationships. Therapeutic target research (green), Prognostic research (orange), and Mechanism research (purple) are three major clusters in TNBC research. The circle size represents the number of publications on each topic; the line’s thickness represents the weight of the connection between each topic.

The topic network analyzed by LDA and Louvain algorithm highlights the areas where interrelated topic clusters appear simultaneously and provides remarkable insights into the relationships between the essential topics of interest. We divided publications into 50 topics. The results of the LDA analysis suggest that all TNBC-related studies are mainly focused on three clusters, i.e., Therapeutic target research, Prognostic research, and Mechanism research (Figure 5). However, few studies on hospice care, patient perspective, surgical treatment of metastasis, and economics are available.

The Therapeutic target research cluster contains 3,465 publications. The research focuses on Therapeutic target research, Protein expression, and Chemotherapy research. This cluster is particularly close to the other two clusters, indicating that the relationship between essential clinical integration and TNBC basic research is very close. We also found that clinical trials can quickly transform basic research into clinical practice to improve patient prognosis.

In the Prognostic research cluster, Survival related research and Demography research are the most studied topics. There are 1,275 publications on Prognostic research, which account for the most significant proportion and are closely related to the other two topics, indicating that prognostic research is the research focus. Interestingly, we found that Demography research and Methylation research are highly connected, weighing 359. We further analyzed and found that TNBC methylation differs significantly among races with different genetic backgrounds, and long-term survival studies are lacking.

In the Mechanism research cluster, we found that Apoptosis research, Growth factors study, and Nanoparticle research are the three most researched topics. In addition, The research cluster contains 21 topics, accounting for up to 42%, covering everything from basic medical research to clinical research.

### 3.6. LDA results: Therapeutic target research and Nanoparticle research are the research focus

**FIGURE 6:**
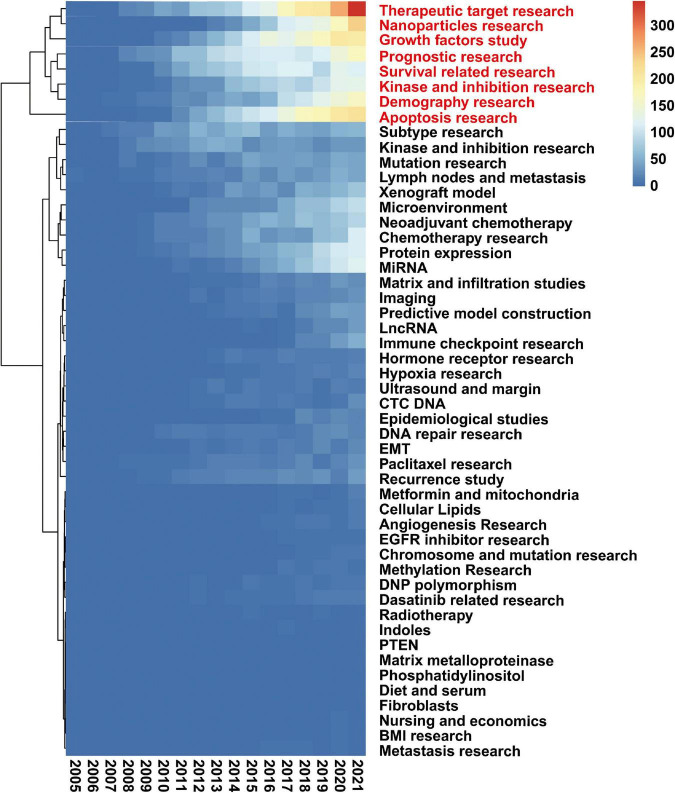
Therapeutic target research and Nanoparticles research are research focus. Heatmap presents the change of 50 research topics of triple-negative breast cancer (TNBC). Latent Dirichlet Allocation (LDA) generated all data. The topics marked in red are the research focus. The lighter the color in the figure, the more publications.

To understand the changes in research focus, we visualized the LDA results and generated a heat map showing the changes in all 50 research topics of TNBC obtained by the LDA algorithm (Figure 6). The number of publications on therapeutic target research and nanoparticle research has increased dramatically, with 15.4% and 15.7%. These results indicate these two are research focus in the future.

### 3.7. LDA and citation analysis results: TNBC research is based on technology that advances TNBC subtyping, new drug development, and clinical trials

Highly cited publications often represent the emergence of outstanding contributions, leading knowledge, or examples in the field. Attention was paid to the citations of publications within the TNBC field. All publications with a total of 490,599 citations, among which the top ten publications with the highest internal citations are listed in Table 3, the publication with the highest internal citations, 1,293, and the total citations of these 10 publications are 21,550. These publications focus on three categories, clinical characteristics of extensive population studies (15–17), clinical trials of new medications (18–21), and subtyping studies of TNBC (22–24). They represent researchers focused on discovering new molecular targets and developing multiple therapies such as Atezolizumab and Nab-Paclitaxel for treatment. Therefore, under the guidance of this research model, similar studies in the future can get more citations. On the other hand, combined with the steady increase of MeSH terms year by year, the lack of drastic changes suggests that TNBC research presents a stable and mature research model, that is, new drug development based on TNBC typing, target drug development, and clinical trials.

**TABLE 3:** Top 10 publications of triple-negative breast cancer (TNBC) based on internal citations and Latent Dirichlet Allocation (LDA) results.

Reference title DOI	References	Internal citation	Total citation	LDA results
J Clin Invest. 2011 Jul; 121 (7): 2750-67 https://doi.org/10.1172/jci45014	Identification of human triple-negative breast cancer subtypes and preclinical models for selection of targeted therapies (24)	1,293	3,205	Protein expression
Clin Cancer Res. 2007 Aug 1; 13 (15 Pt 1): 4429-34 https://doi.org/10.1158/1078-0432.ccr-06-3045	Triple-negative breast cancer: clinical features and patterns of recurrence (23)	1,220	3,025	Subtype research
N Engl J Med. 2010 Nov 11; 363 (20): 1938-48 https://doi.org/10.1056/nejmra1001389	Triple-negative breast cancer (21)	1,062	2,501	Therapeutic target research
J Clin Oncol. 2008 Mar 10; 26 (8): 1275-81 https://doi.org/10.1200/jco.2007.14.4147	Response to neoadjuvant therapy and long-term survival in patients with triple-negative breast cancer (20)	700	1,909	Prognostic research
Cancer. 2007 May 1; 109 (9): 1721-8 https://doi.org/10.1002/cncr.22618	Descriptive analysis of estrogen receptor (ER)-negative, progesterone receptor (PR)-negative, and HER2-negative invasive breast cancer, the so-called triple-negative phenotype: a population-based study from the California cancer Registry (17)	550	1,483	Demography research
Clin Cancer Res. 2007 Apr 15; 13 (8): 2329-34 https://doi.org/10.1158/1078-0432.ccr-06-1109	The triple negative paradox: primary tumor chemosensitivity of breast cancer subtypes (16)	515	1,472	Subtype research
Nat Rev Clin Oncol. 2016 Nov; 13 (11): 674-690 https://doi.org/10.1038/nrclinonc.2016.66	Triple-negative breast cancer: challenges and opportunities of a heterogeneous disease (15)	485	1,280	Therapeutic target research
N Engl J Med. 2018 Nov 29; 379 (22): 2108-2121 https://doi.org/10.1056/nejmoa1809615	Atezolizumab and Nab-Paclitaxel in Advanced Triple-Negative Breast Cancer (19)	358	2,064	Immune checkpoint research
Lancet. 2014 Jul 12;384 (9938): 164-72 https://doi.org/10.1016/s0140-6736(13)62422-8	Pathological complete response and long-term clinical benefit in breast cancer: the CTNeoBC pooled analysis (18)	335	2,113	Neoadjuvant chemotherapy
Ann Oncol. 2011 Aug; 22 (8): 1736-47 https://doi.org/10.1093/annonc/mdr304	Strategies for subtypes–dealing with the diversity of breast cancer: highlights of the St. Gallen International Expert Consensus on the Primary Therapy of Early Breast Cancer 2011 (22)	311	2,498	Therapeutic target research

## 4. Discussion

We analyzed 16,826 publications in the field of TNBC from 2005 to 2022 using machine learning and NLP. Furthermore, we visualize and analyze the results from a macro perspective. Over the past 17 years, we found that TNBC-related publications have increased from none to 16,826 in 2021, with more extensive research content. TNBC research focuses on Therapeutic target research, Prognostic research, and Mechanism research. Research topics have changed over the years, and the current research focus is expected to be Therapeutic target research and Nanoparticle research, according to our LDA results.

Bibliometrics is a compelling analysis method to obtain information from massive texts quantitatively, and there are very few bibliometrics analyses on TNBC such as VOSviewer, Bibliographic Items Co-occurrence Matrix Builder (BICOMB), and CiteSpace. However, with the development of the publishing industry, these tools have difficulty applying to massive publication analysis due to their architecture, insufficient computer memory, and sharing protocols. Therefore, our research uses the LDA algorithm based on Python, an unsupervised topic model. Furthermore, our topic model is based on the publication’s abstract, not on the keywords. It is easy to use with negligible memory consumption and can analyze massive publications.

We found that Therapeutic target research has always been research-focused because TNBC lacks effective therapeutic targets and has high heterogeneity (24, 25). Our research found that this part contains a variety of attempts, DNA repair research, immune checkpoint research, and protein expression. We only found 137 publications related to immune checkpoint research, and immunotherapy research is not closely related to the prognosis and mechanism research of TNBC. Several clinical studies are being carried out, including IMpassion130, KEYNOTE-355, and Impassion 131 (26–28). Some positive results can reduce the risk of death by up to 35%. However, more important is the research on the underlying mechanism and the exploration of various influencing factors, especially the extracellular matrix, hypoxia, and immune cell infiltration (29). In addition, immune checkpoint research has just started for five years, according to our results, and several medications have already been applied in the clinic. This research trend will continue, and immunotherapy will become a safe and effective treatment option.

The research scope of the TNBC mechanism is pervasive, covering the immune microenvironment and subtypes of TNBC. The successful subtyping provides a solid theoretical basis for the precision therapy of TNBC (30). Gene sequencing technology allows us to fully understand the mutation rate of TNBC, which is about 1.68 bp/Mb (31). Mutations occur in genes in multiple key signaling pathways such as PI3K/Akt/mTOR pathway, RAS/RAF/MEK pathway, JAK/STAT pathway, DNA repair pathway, and cell cycle checkpoint (32–34). Therefore, various treatments targeting the signal pathways are currently undergoing clinical trials. Some inhibitors have been used as potential medications for TNBC treatment, including PI3K, MEK, PARP, EGFR, VEGF, and AR inhibitors (32).

Triple-negative breast cancer subtyping has always been the focus of research. There is no unified standard based on the TNBC genome and cell heterogeneity. The first classification was based on Lehmann’s gene expression analysis of breast cancer and constructed a “triple negative classification” and six subclassifications (24). In 2016, Lehmann’s further research found that immunomodulatory (IM) patients are more likely to benefit from checkpoint inhibitor therapy (35). With the advancement of technology, such as the emergence of single-cell RNA sequencing, spatial transcriptomics, and radionics, and the further expansion of data volume, new technologies have provided new insights into the typing of TNBC and proposed guidance for treatment. Xie’s research established a new prognostic model through the comprehensive analysis of multiple cell death patterns on more than 1,000 breast cancer patients, which can predict the clinical prognosis and drug sensitivity after TNBC surgery (36). In addition to technological progress, an in-depth understanding of the oncological course, mechanism of occurrence and development, and algorithm advances will provide a more detailed classification of TNBC.

On the other hand, studies on operations and radiotherapy were rarely reported, especially for re-operations related to local-regional recurrence risk or distant metastasis. Many studies suggest that surgery is essential in treating distant metastases of cancers, such as colorectal cancer (37). In addition, many studies on other cancers, including pancreatic and colorectal cancer, demonstrated that the tumor microenvironment, especially the extracellular matrix, has been found to play an essential role in cancer metastasis, local recurrence, and chemotherapeutic drug resistance (38, 39). Many potential drugs are used due to their ability to target the extracellular matrix, such as PEGPH20 (an enzyme that targets matrix hyaluronic acid), pegilodecakin (a PEGylated IL-10) (40, 41). However, the study on extracellular matrix in TNBC is insufficient so far.

Although the research on TNBC has made significant progress in many aspects, the present research also found some research deficiencies on TNBC. There is a lack of research on TNBC from patients’ perspectives, health economics, and hospice care. Although, at present, the 5 years overall survival rate of most tumors has been dramatically improved, helping tumor patients with psychological issues re-enter society will become a new important research topic (42). TNBC patients are more likely to relapse and metastasize than other breast cancer subtypes, resulting in more significant mental and economic pressure on patients and their families. Studies on patients with more prolonged survival can better understand TNBC and even other long-term survival tumors (43). In the future, we will face more challenges for patients with a long survival period of 5–10 years (44).

There are some limitations in the present study. Besides PubMed, several other databases, including Scopus, Web of Science, and Embase, could be used for bibliometric research. Although PubMed contains the highest quality peer-reviewed research and excludes irrelevant, non-peer-reviewed publications, the literature will provide detailed and comprehensive knowledge if other databases are explored simultaneously. Secondly, we considered that all publications publish more positive research results. Negative results and clinical participants’ perspectives are naturally more difficult to be published. With the development of complete medical record texts, publication databases, and improved algorithms, it is reasonable for machine learning to play a more active auxiliary role in future clinical practice. The data presented in this study will hopefully help scientists understand the current status of TNBC research and design more relevant basic and clinical research projects.

## 5. Conclusion

We analyzed 16,826 TNBC publications through the NLP Method. TNBC research shows insufficiencies, especially in long-term survival-related research, and a lack of research from patients’ perspectives. The publications mainly focused on three aspects: Therapeutic target research, Prognostic research, and Mechanism research. The research direction of TNBC may require the intervention of new technologies.

## Data availability statement

The original contributions presented in this study are included in the article/Supplementary material, further inquiries can be directed to the corresponding authors.

## Author contributions

KW initiated the project, analyzed the data, constructed analytical methods, and wrote the primary manuscript draft. XD initiated and supervised all aspects of the project and wrote the primary manuscript draft. CZ performed statistical analyses and contributed to the manuscript writing. DD helped interpret results and contributed to the statistical analyses. LZ contributed to the manuscript’s revision in terms of writing and interpretation. ML contributed to the interpreting results and supervising statistical analyses. All authors contributed to the manuscript writing and read and approved the final version of the manuscript.

## Footnotes

1
https://pubmed.ncbi.nlm.nih.gov/

2 https://www.r-project.org/, version:4.1.1

3 https://www.python.org/, version 3.7.1

4
https://cran.r-project.org/web/packages/easyPubMed/index.html

5
https://github.com/christopherBelter/pubmedXML

6
https://github.com/mxdwangdali11/guid-to-Bibliometric-LDA-Analysis

7
https://doi.org/10.5281/zenodo.7461925

8 https://gephi.org/, version 0.9.2

## References

[1] Banerjee S Tian T Wei Z Shih N Feldman MD Peck KN Distinct microbial signatures associated with different breast cancer types. *Front Microbiol.* (2018) 9:951. 10.3389/fmicb.2018.00951 29867857 PMC5962706

[2] Perou CM Sorlie T Eisen MB van de Rijn M Jeffrey SS Rees CA Molecular portraits of human breast tumours. *Nature.* (2000) 406:747–52. 10.1038/35021093 10963602

[3] Pareja F Reis-Filho JS. Triple-negative breast cancers - a panoply of cancer types. *Nat Rev Clin Oncol.* (2018) 15:347–8. 10.1038/s41571-018-0001-7 29555966

[4] Yi H Wu M Zhang Q Lu L Yao H Chen S Reversal of HER2 negativity: an unexpected role for lovastatin in triple-negative breast cancer stem cells. *J Cancer.* (2020) 11:3713–6. 10.7150/jca.39265 32328175 PMC7171481

[5] Tran BX Latkin CA Sharafeldin N Nguyen K Vu GT Tam WWS Characterizing artificial intelligence applications in cancer research: a latent dirichlet allocation analysis. *JMIR Med Inform.* (2019) 7:e14401. 10.2196/14401 31573929 PMC6774235

[6] Teles RHG Moralles HF Cominetti MR. Global trends in nanomedicine research on triple negative breast cancer: a bibliometric analysis. *Int J Nanomedicine.* (2018) 13:2321–36. 10.2147/IJN.S164355 29713164 PMC5910795

[7] Buchlak QD Esmaili N Leveque JC Farrokhi F Bennett C Piccardi M Machine learning applications to clinical decision support in neurosurgery: an artificial intelligence augmented systematic review. *Neurosurg Rev.* (2020) 43:1235–53. 10.1007/s10143-019-01163-8 31422572

[8] Jun I Rich SN Chen Z Bian J Prosperi M. Challenges in replicating secondary analysis of electronic health records data with multiple computable phenotypes: A case study on methicillin-resistant staphylococcus aureus bacteremia infections. *Int J Med Inform.* (2021) 153:104531. 10.1016/j.ijmedinf.2021.104531 34332468 PMC8451470

[9] Feng C Wu Y Gao L Guo X Wang Z Xing B. Publication landscape analysis on gliomas: how much has been done in the past 25 years? *Front Oncol.* (2019) 9:1463. 10.3389/fonc.2019.01463 32038995 PMC6988829

[10] Li C Liu Z Shi R. A bibliometric analysis of 14,822 researches on myocardial reperfusion injury by machine learning. *Int J Environ Res Public Health.* (2021) 18:8231. 10.3390/ijerph18158231 34360526 PMC8345983

[11] Wang K Feng C Li M Pei Q Li Y Zhu H A bibliometric analysis of 23,492 publications on rectal cancer by machine learning: basic medical research is needed. *Therap Adv Gastroenterol.* (2020) 13:1756284820934594. 10.1177/1756284820934594 32782478 PMC7385823

[12] Kumar R Rani S Awadh MA. Exploring the application sphere of the internet of things in industry 4.0: a review, bibliometric and content analysis. *Sensors.* (2022) 22:4276. 10.3390/s22114276 35684897 PMC9185372

[13] Kumar R Goel P. Exploring the domain of interpretive structural modelling (ism) for sustainable future panorama: a bibliometric and content analysis. *Arch Comput Methods Eng.* (2022) 29:2781–810. 10.1007/s11831-021-09675-7

[14] Traag VA. Faster unfolding of communities: speeding up the louvain algorithm. *Phys Rev E Stat Nonlin Soft Matter Phys.* (2015) 92:032801. 10.1103/PhysRevE.92.032801 26465522

[15] Bianchini G Balko JM Mayer IA Sanders ME Gianni L. Triple-negative breast cancer: challenges and opportunities of a heterogeneous disease. *Nat Rev Clin Oncol.* (2016) 13:674–90. 10.1038/nrclinonc.2016.66 27184417 PMC5461122

[16] Carey LA Dees EC Sawyer L Gatti L Moore DT Collichio F The triple negative paradox: primary tumor chemosensitivity of breast cancer subtypes. *Clin Cancer Res.* (2007) 13:2329–34. 10.1158/1078-0432.CCR-06-1109 17438091

[17] Bauer KR Brown M Cress RD Parise CA Caggiano V. Descriptive analysis of estrogen receptor (ER)-negative, progesterone receptor (PR)-negative, and HER2-negative invasive breast cancer, the so-called triple-negative phenotype: a population-based study from the California cancer registry. *Cancer.* (2007) 109:1721–8. 10.1002/cncr.22618 17387718

[18] Cortazar P Zhang L Untch M Mehta K Costantino JP Wolmark N Pathological complete response and long-term clinical benefit in breast cancer: the CTNeoBC pooled analysis. *Lancet.* (2014) 384:164–72. 10.1016/S0140-6736(13)62422-8 24529560

[19] Schmid P Adams S Rugo HS Schneeweiss A Barrios CH Iwata H Atezolizumab and nab-paclitaxel in advanced triple-negative breast cancer. *N Engl J Med.* (2018) 379:2108–21. 10.1056/NEJMoa1809615 30345906

[20] Liedtke C Mazouni C Hess KR Andre F Tordai A Mejia JA Response to neoadjuvant therapy and long-term survival in patients with triple-negative breast cancer. *J Clin Oncol.* (2008) 26:1275–81. 10.1200/JCO.2007.14.4147 18250347

[21] Foulkes WD Smith IE Reis-Filho JS. Triple-negative breast cancer. *N Engl J Med.* (2010) 363:1938–48. 10.1056/NEJMra1001389 21067385

[22] Goldhirsch A Wood WC Coates AS Gelber RD Thurlimann B Senn HJ Strategies for subtypes–dealing with the diversity of breast cancer: highlights of the st. gallen international expert consensus on the primary therapy of early breast cancer 2011. *Ann Oncol.* (2011) 22:1736–47. 10.1093/annonc/mdr304 21709140 PMC3144634

[23] Dent R Trudeau M Pritchard KI Hanna WM Kahn HK Sawka CA Triple-negative breast cancer: clinical features and patterns of recurrence. *Clin Cancer Res.* (2007) 13:4429–34. 10.1158/1078-0432.CCR-06-3045 17671126

[24] Lehmann BD Bauer JA Chen X Sanders ME Chakravarthy AB Shyr Y Identification of human triple-negative breast cancer subtypes and preclinical models for selection of targeted therapies. *J Clin Invest.* (2011) 121:2750–67. 10.1172/JCI45014 21633166 PMC3127435

[25] Deng X Faqing T Rosol TJ. *Triple-Negative Breast Cancer.* Singapore: World Scientific (2020). p. 21–70. 10.1142/11199

[26] Cortes J Cescon DW Rugo HS Nowecki Z Im SA Yusof MM Pembrolizumab plus chemotherapy versus placebo plus chemotherapy for previously untreated locally recurrent inoperable or metastatic triple-negative breast cancer (KEYNOTE-355): a randomised, placebo-controlled, double-blind, phase 3 clinical trial. *Lancet.* (2020) 396:1817–28. 10.1200/JCO.2020.38.15_suppl.1000 33278935

[27] Miles D Gligorov J Andre F Cameron D Schneeweiss A Barrios C Primary results from IMpassion131, a double-blind, placebo-controlled, randomised phase III trial of first-line paclitaxel with or without atezolizumab for unresectable locally advanced/metastatic triple-negative breast cancer. *Ann Oncol.* (2021) 32:994–1004. 10.1016/j.annonc.2020.08.2243 34219000

[28] Emens LA Adams S Barrios CH Dieras V Iwata H Loi S First-line atezolizumab plus nab-paclitaxel for unresectable, locally advanced, or metastatic triple-negative breast cancer: IMpassion130 final overall survival analysis. *Ann Oncol.* (2021) 32:983–93. 10.1016/j.annonc.2021.05.355 34272041

[29] Bou-Dargham MJ Draughon S Cantrell V Khamis ZI Sang QA. Advancements in human breast cancer targeted therapy and immunotherapy. *J Cancer.* (2021) 12:6949–63. 10.7150/jca.64205 34729098 PMC8558657

[30] Lee YM Oh MH Go JH Han K Choi SY. Molecular subtypes of triple-negative breast cancer: understanding of subtype categories and clinical implication. *Genes Genomics.* (2020) 42:1381–7. 10.1007/s13258-020-01014-7 33145728

[31] Mittendorf EA Philips AV Meric-Bernstam F Qiao N Wu Y Harrington S PD-L1 expression in triple-negative breast cancer. *Cancer Immunol Res.* (2014) 2:361–70. 10.1158/2326-6066.CIR-13-0127 24764583 PMC4000553

[32] Islam R Lam KW. Recent progress in small molecule agents for the targeted therapy of triple-negative breast cancer. *Eur J Med Chem.* (2020) 207:112812. 10.1016/j.ejmech.2020.112812 32937283

[33] Shah SP Roth A Goya R Oloumi A Ha G Zhao Y The clonal and mutational evolution spectrum of primary triple-negative breast cancers. *Nature.* (2012) 486:395–9. 22495314 10.1038/nature10933 PMC3863681

[34] Vanhaesebroeck B Guillermet-Guibert J Graupera M Bilanges B. The emerging mechanisms of isoform-specific PI3K signalling. *Nat Rev Mol Cell Biol.* (2010) 11:329–41. 10.1038/nrm2882 20379207

[35] Lehmann BD Jovanovic B Chen X Estrada MV Johnson KN Shyr Y Refinement of triple-negative breast cancer molecular subtypes: implications for neoadjuvant chemotherapy selection. *PLoS One.* (2016) 11:e0157368. 10.1371/journal.pone.0157368 27310713 PMC4911051

[36] Zou Y Xie J Zheng S Liu W Tang Y Tian W Leveraging diverse cell-death patterns to predict the prognosis and drug sensitivity of triple-negative breast cancer patients after surgery. *Int J Surg.* (2022) 107:106936. 10.1016/j.ijsu.2022.106936 36341760

[37] Dijkstra M Nieuwenhuizen S Puijk RS Timmer FEF Geboers B Schouten EAC Primary tumor sidedness, ras and braf mutations and msi status as prognostic factors in patients with colorectal liver metastases treated with surgery and thermal ablation: results from the amsterdam colorectal liver met registry (AmCORE). *Biomedicines.* (2021) 9:962. 10.3390/biomedicines9080962 34440165 PMC8395017

[38] Gu Z Du Y Zhao X Wang C. Tumor microenvironment and metabolic remodeling in gemcitabine-based chemoresistance of pancreatic cancer. *Cancer Lett.* (2021) 52:98–108. 10.1016/j.canlet.2021.08.029 34461181

[39] Song X Xie D Tan F Zhou Y Li Y Zhou Z Intravascular emboli relates to immunosuppressive tumor microenvironment and predicts prognosis in stage III colorectal cancer. *Aging.* (2021) 13:20609–28. 10.18632/aging.203451 34438367 PMC8436899

[40] Gourd E. PEGPH20 for metastatic pancreatic ductal adenocarcinoma. *Lancet Oncol.* (2018) 19:e81. 10.1016/S1470-2045(17)30953-1 29276021

[41] Hecht JR Lonardi S Bendell J Sim HW Macarulla T Lopez CD Randomized phase iii study of folfox alone or with pegilodecakin as second-line therapy in patients with metastatic pancreatic cancer that progressed after gemcitabine (SEQUOIA). *J Clin Oncol.* (2021) 39:1108–18. 10.1200/JCO.20.02232 33555926 PMC8078437

[42] Watkins CC Kanu IK Hamilton JB Kozachik SL Gaston-Johansson F. Differences in coping among African American women with breast cancer and triple-negative breast cancer. *Oncol Nurs Forum.* (2017) 44:689–702. 10.1188/17.ONF.689-702 29052667

[43] Mediratta K El-Sahli S D’Costa V Wang L. Current progresses and challenges of immunotherapy in triple-negative breast cancer. *Cancers.* (2020) 12:3529. 10.3390/cancers12123529 33256070 PMC7761500

[44] Ertas G Basal FB Ucer AR Benzer E Altundag MB Demirci U Clinical features of metaplastic breast carcinoma: A single-center experience. *J Cancer Res Ther.* (2020) 16:1229–34. 33342778 10.4103/jcrt.JCRT_964_19

