# Supplementary material for: A bibliometric analysis of 16,826 triple-negative breast cancer publications using multiple machine learning algorithms: Progress in the past 17 years
Source: Front Med (Lausanne). 2023 Feb 8;10:999312. doi: 10.3389/fmed.2023.999312 (PMC9945529; doi:10.3389/fmed.2023.999312)
Supplement: Supplementary file 1 [file Data_Sheet_1.docx]

**Supplement**

**A bibliometric analysis of 16,826 triple-negative breast cancer (TNBC) publications using multiple machine learning algorithms: Progress in the past 17 years**

Kangtao Wang, Chanjuan Zheng, Lian Xue, Dexin Deng, Liang Zeng, Ming Li, Xiyun Deng

**Table of Content**

**Table S1.**Top 10 journals with the most publications

**Table S2.** Most widely studied top 20 MeSH terms in TNBC research

**Supplemental information 1** Step-by-step instruction and core codes

**Supplemental information 1** Top 10 journals with the most publications

| **Journal** | **No. publications** |
| --- | --- |
| **Breast Cancer Res Treat** | 690 |
| **PLoS One** | 427 |
| **Sci Rep** | 331 |
| **Cancers (Basel)** | 308 |
| **BMC Cancer** | 277 |
| **Breast Cancer Res** | 276 |
| **Front Oncol** | 214 |
| **Clin Cancer Res** | 212 |
| **Cancer Res** | 203 |
| **Int J Mol Sci** | 180 |

**Table S1** Most widely studied top 20 MeSH terms in TNBC research

| **Mesh terms** | **Number of indicated MeSH terms/years** | | | | |
| --- | --- | --- | --- | --- | --- |
|  | **1995-2009** | **2010-2014** | **2015-2019** | **2020-2021** | **Total** |
| **pathology** | 231 | 1,934 | 4,215 | 1,566 | 7,946 |
| **metabolism** | 206 | 1,904 | 3,606 | 1,464 | 7,180 |
| **genetics** | 111 | 1,239 | 2,921 | 1,237 | 5,508 |
| **drug therapy** | 111 | 972 | 2,328 | 1,232 | 4,643 |
| **Cell Line, Tumor** | 28 | 704 | 2,495 | 1,141 | 4,368 |
| **pharmacology** | 28 | 507 | 1,720 | 889 | 3,144 |
| **drug effects** | 23 | 469 | 1,768 | 752 | 3,012 |
| **Prognosis** | 90 | 808 | 1,389 | 563 | 2,850 |
| **therapeutic use** | 87 | 672 | 1,322 | 612 | 2,693 |
| **Biomarkers, Tumor** | 107 | 729 | 1,323 | 511 | 2,670 |
| **Receptor, ErbB-2** | 179 | 1,113 | 887 | 288 | 2,467 |
| **Gene Expression Regulation, Neoplastic** | 28 | 454 | 1,334 | 559 | 2,375 |
| **Cell Proliferation** | 15 | 332 | 1,282 | 615 | 2,244 |
| **Antineoplastic Agents** | 46 | 444 | 1,105 | 453 | 2,048 |
| **Receptors, Estrogen** | 185 | 1,021 | 625 | 166 | 1,997 |
| **methods** | 41 | 313 | 1,136 | 451 | 1,941 |
| **mortality** | 68 | 543 | 945 | 290 | 1,846 |
| **chemistry** | 55 | 355 | 982 | 441 | 1,833 |
| **Receptors, Progesterone** | 179 | 924 | 479 | 126 | 1,708 |
| **Neoplasm Staging** | 51 | 500 | 712 | 196 | 1,459 |

**Supplemental information 1** Step by step instruction

**Step 1: Confirmation of research purpose**

To identify the purpose of the research, this study uses machine learning methods to explore the current research status and deficiencies from a macro perspective on TNBC publications. Then based on the research purpose, select the appropriate database and search terms, search steps, and inclusion and exclusion criteria. We describe the specific details in Table 1.

**Table 1. TNBC publications assortment steps.**

| Exploration Steps | Query on PubMed | Description |
| --- | --- | --- |
| 1 | Depression | ("triple negative breast neoplasms"[MeSH Terms] OR ("triple"[All Fields] AND "negative"[All Fields] AND "breast"[All Fields] AND "neoplasms"[All Fields]) OR "triple negative breast neoplasms"[All Fields]) |
| 2 | Data limiation | (2005:2022[pdat]) |

**Step2：Publication access**

Confirm the search strategy, the database, and the API used based on the research purpose. This study uses the PubMed database https://pubmed.ncbi.nlm.nih.gov/ to obtain as many publications about TNBC as possible, and the download method R were used.

The retrieval code is as follows:

setwd("")

library(easyPubMed)

myQuery <- '("triple negative breast neoplasms"[MeSH Terms] OR ("triple"[All Fields] AND "negative"[All Fields] AND "breast"[All Fields] AND "neoplasms"[All Fields]) OR "triple negative breast neoplasms"[All Fields])triple negative breast cancer AND ("2005/01/01"[PDAT] : "2022/01/01"[PDAT])'

myIdList <- get_pubmed_ids(myQuery)

fdt_files <- batch_pubmed_download(pubmed_query_string = myQuery,

format = "xml",

batch_size = 4000,

dest_file_prefix = "fdt",

encoding = "UTF-8");

fdt_list <- lapply(fdt_files, table_articles_byAuth,

included_authors = "last", getKeywords = TRUE)

**Step 3: Inclusion and exclusion criteria**

After obtaining publications, the required publications were further included and excluded. Repeat steps 1 and 2 until it fits research purpose. We put our inclusion and exclusion criteria in Table 2.

**Table 2. Inclusive and Exclusive Criteria**

| **Parameter of Selection of a publication** | **Inclusion criterion** | **Exclusion criterion** | **The rationale for Inclusion–Exclusion** |
| --- | --- | --- | --- |
| Language | English | Other languages | The working language of the LDA algorithm is English. Other languages are not recognized |
| Publication date | 2005–2021 | Publications before 2021 and after 2005 | Not included in the 2022 publication as it has not been fully published |
| Publication Type | All | Missing data, Meeting abstract, Proceeding paper, Book review, News item | As the LDA algorithm is unsupervised machine learning, the analysis must include abstract as the text editor. In addition to incomplete content, try to include research articles and reviews. |
| Funding Sponsor | All | No Exclusion | This parameter does not affect the selection criterion |
| Affiliation/ Organization | All | No Exclusion | This parameter does not affect the selection criterion |
| Funding | All | No Exclusion | This parameter does not affect the selection criterion |
| Country | All | No Exclusion | Publication from each country has its significance |

**Step 4：General literature information studies**

We have attached a partial example of downloading the original file in the file. Each publication in the study included year; title; Abstract; Author; Affiliation; Country; MedlineTA; Substance; CitationSubset; MeshHeadingList; Reference_ArticleId; Reference_title; Publication_Type; received_date; accepted_date; pubmed_date; medline_date; entrez_date; revised_date. Based on the above information, data extraction and basic literature information analysis can be carried out. Because the original content is too large, we only use it here as an example. For details, please refer to the supplementary materials we uploaded, and Figure 1 is the original data.


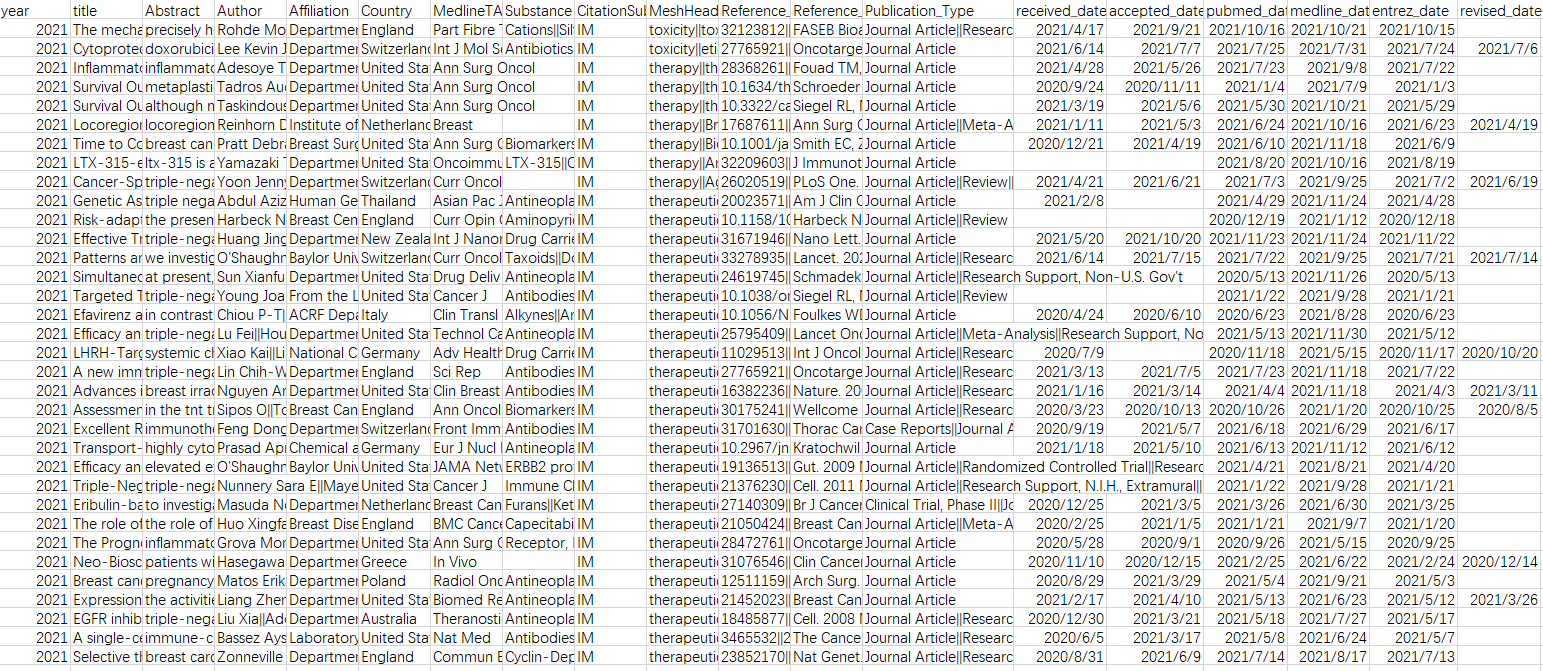


**Figure 1. raw data example**

**Step 5: Topic Modeling, LDA Analysis**

The LDA algorithm is an unsupervised text analysis algorithm and a text topic model [1]。LDA is a very typical bag of words model. Based on the example theory, each document is a collection of phrases, and there is no order or sequence relationship between words. A document can contain multiple topics, and each word in the document is generated by one of the topics. Based on this way of thinking, as long as you use our algorithm, you can get the result. We use Python, and you can directly use the sample data we uploaded for analysis. The specific code is as follows:

LDA code:

from ast import Try

from nltk.tokenize import RegexpTokenizer

#from stop_words import get_stop_words

from nltk.stem.porter import PorterStemmer

from sklearn.utils import shuffle

from gensim import corpora, models

import pandas as pd

import logging

import pickle

import numpy as np

import os,sys

from matplotlib import pyplot as plt

logging.basicConfig(level = logging.INFO,format = '%(asctime)s - %(name)s - %(levelname)s - %(message)s')

logger = logging.getLogger(__name__)

from gensim.models.ldamulticore import LdaMulticore

def dumppick(filepath,Year):

corpus = []

tokens = []

df = pd.read_csv(filepath,sep='\t',encoding="utf-8-sig",error_bad_lines=False)

df = df[df["Abstract"].isna()!=True]

df.astype({'year': 'int32'})

df = df[df.year<Year]

for line in df["Abstract"]:

corpus.append(line.strip())

del df

en_stop = ['i', 'me', 'my', 'myself', 'we', 'our', 'ours', 'ourselves', 'you', 'your', 'yours', 'yourself', 'yourselves', 'he', 'him', 'his', 'himself', 'she', 'her', 'hers', 'herself', 'it', 'its', 'itself', 'they', 'them', 'their', 'theirs', 'themselves', 'what', 'which', 'who', 'whom', 'this', 'that', 'these', 'those', 'am', 'is', 'are', 'was', 'were', 'be', 'been', 'being', 'have', 'has', 'had', 'having', 'do', 'does', 'did', 'doing', 'a', 'an', 'the', 'and', 'but', 'if', 'or', 'because', 'as', 'until', 'while', 'of', 'at', 'by', 'for', 'with', 'about', 'against', 'between', 'into', 'through', 'during', 'before', 'after', 'above', 'below', 'to', 'from', 'up', 'down', 'in', 'out', 'on', 'off', 'over', 'under', 'again', 'further', 'then', 'once', 'here', 'there', 'when', 'where', 'why', 'how', 'all', 'any', 'both', 'each', 'few', 'more', 'most', 'other', 'some', 'such', 'no', 'nor', 'not', 'only', 'own', 'same', 'so', 'than', 'too', 'very', 's', 't', 'can', 'will', 'just', 'don', 'should', 'now', 'd', 'll', 'm', 'o', 're', 've', 'y', 'ain', 'aren', 'couldn', 'didn', 'doesn', 'hadn', 'hasn', 'haven', 'isn', 'ma', 'mightn', 'mustn', 'needn', 'shan', 'shouldn', 'wasn', 'weren', 'won', 'wouldn']

p_stemmer = PorterStemmer()

logging.info("wenbenyuchuli")

tokenizer = RegexpTokenizer(r'[A-Za-z]+')

for i,text in enumerate(corpus):

if i%1000==0:

logging.info(f"{i} line done")

raw = text.lower()

token = tokenizer.tokenize(raw)

stop_remove_token = [word for word in token if (word not in en_stop and len(word)>1)]

stem_token = [p_stemmer.stem(word) for word in stop_remove_token]

tokens.append(stem_token)

# tokens.append(token)

# print tokens

logging.info("start")

logging.info("basic")

dictionary = corpora.Dictionary(tokens)

logging.info("word bags")

texts = [dictionary.doc2bow(text) for text in tokens]

logging.info("finished")

logging.info("start tfidf")

texts_tf_idf = models.TfidfModel(texts)[texts]

pickle.dump(texts, open("text_dtm.pickle","wb"))

pickle.dump(texts_tf_idf, open("texts_tf_idf_dtm.pickle","wb"))

pickle.dump(dictionary, open("dictionary.pickle","wb"))

def loadpcik():

texts = pickle.load(open("text_dtm.pickle","rb"))

texts_tf_idf = pickle.load(open("texts_tf_idf_dtm.pickle","rb"))

dictionary = pickle.load(open("dictionary.pickle","rb"))

return texts, texts_tf_idf,dictionary

#dumppick()

def calc_n_of_lda(filename,Year,start,end):

dumppick(filename,Year)

texts, texts_tf_idf, dictionary = loadpcik()

"""

print("**************LSI*************")

lsi = models.lsimodel.LsiModel(corpus=texts, id2word=dictionary, num_topics=20)

texts_lsi = lsi[texts_tf_idf]

print(lsi.print_topics(num_topics=20, num_words=10))

"""

logging.info("**************LDA*************")

ppl = []

for num_topics in range(start,end,1):

texts = shuffle(texts)

lda = LdaMulticore(corpus=texts,iterations=50, id2word=dictionary, num_topics=num_topics,passes=10,per_word_topics=True)

#texts_lda = lda[texts_tf_idf]

# print(lda.print_topics(num_topics=num_topics, num_words=10),file =out)

# ppl.append(np.exp2(-lda.log_perplexity(texts))

ppl.append(lda.log_perplexity(texts))

plt.plot( range(10,60,1),ppl)

plt.title("num_topics(x) - perplexity(y)")

plt.savefig("prop.png")

plt.show()

return lda, texts, texts_tf_idf, dictionary, ppl

def load_lda(filename,num_topics):

texts, texts_tf_idf, dictionary = loadpcik()

lda = LdaMulticore(corpus=texts,iterations=100, id2word=dictionary, num_topics=num_topics,passes=20,per_word_topics=True)

lda.save("./ldamd/{}tpc+{}".format(num_topics,filename[9:18]))

return lda, texts, texts_tf_idf, dictionary,

def saveldatpcw(lda,num_topics):

tpcn = num_topics

tpcw = pd.DataFrame(columns=[i for i in range(1,11)])

for i in range(tpcn):

tpcw.loc[i] = [ w for w,p in lda.show_topic(i)]

tpcw.to_csv("./newdata/tpcw.csv")

def get_cite_n_dmt(dictionary,citenum=0,):

citenum=0

corpus = []

tokens = []

df = pd.read_csv("pubmed_result_parsed.csv",sep=',',encoding="utf-8-sig")

df = df[df["cite"]==citenum]

df = df[df["Abstract"].isna()!=True]

for line in df["Abstract"]:

corpus.append(line.strip())

del df

en_stop = [ str(i).strip() for i in open("stopwords.txt",encoding="utf-8-sig") ] # erase stop_words

p_stemmer = PorterStemmer()

logging.info("pretreat")

tokenizer = RegexpTokenizer(r'[A-Za-z]+')

for i,text in enumerate(corpus):

if i%1000==0:

print(i)

raw = text.lower()

token = tokenizer.tokenize(raw)

stop_remove_token = [word for word in token if (word not in en_stop and len(word)>1)]

stem_token = [p_stemmer.stem(word) for word in stop_remove_token]

tokens.append(stop_remove_token)

texts_cite_n = [dictionary.doc2bow(text) for text in tokens]

return texts_cite_n

def getallcited2tpc(lda,texts,filename,Year):

tpc1 = []

tpc2 = []

for i in texts:

tpc = lda.get_document_topics(i)

tpc = sorted(tpc,key=lambda x:-x[2])

tpc1.append(tpc[0][0])

if len(tpc)>1:

tpc2.append(tpc[2][0])

else:

tpc2.append(lda.num_topics+1)

df = pd.read_csv(filename,sep='\t',encoding="utf-8-sig")

df = df[df["Abstract"].isna()!=True]

df = df[df.year<Year]

df["tpc1"] = tpc1

df["tpc2"] = tpc2

df.to_csv(filename.replace(".csv","_with_topic.csv"),sep='\t',encoding="utf-8-sig")

return tpc1,tpc2

def grap(tpc1,tpc2,tpcn,filename):

from collections import Counter

CC = Counter(tpc1)

import networkx as nx

G = nx.Graph()

for i in range(tpcn):

G.add_node(i,num=CC[i])

#edgscount = Counter([(i,j) for i,j in zip(tpc1,tpc2)])

#for edgs,count in edgscount.items()

edgeslist = [(i,j) for i,j in zip(tpc1,tpc2) if j<tpcn]

G.add_edges_from(edgeslist,Weight=0)

for i,j in zip(tpc1,tpc2):

if j >= tpcn:

continue

G.edges[i,j]["Weight"]+=1

nx.write_graphml(G,filename.replace(".csv",".graphml"),encoding="utf8")

return G

def Grap_Add_tpcname():

import pandas as pd

c = pd.read_excel("glioblastoma\\topics-gbm.xlsx")

pic = {tpcid:name for tpcid,name in zip( range(50),c["topics"])}

import networkx as nx

g = nx.read_graphml("glioblastoma\\Glioblastoma_50.graphml")

for i in range(50):

g.node[str(i)]["name"] = pic[i]

nx.write_graphml(g,"glioblastoma\\Glioblastoma_50.graphml_addname.graphml",encoding="utf8")

def main(Year):

filename = "total.csv"

if not os.path.exists("./newdata"):

os.mkdir("./newdata")

if not os.path.exists("./ldamd"):

os.mkdir("./ldamd")

start, end = map(int, str(input("Enter calculation subject range comma-separated (example 10,60): ")).split(","))

calc_n_of_lda(filename,Year, start, end)

num_topics = int(input("Enter the number of topics："))

lda, texts, texts_tf_idf, dictionary = load_lda(filename,num_topics)

saveldatpcw(lda,num_topics)

logging.info("LDA finish")

logging.info("in each publication")

tpc1,tpc2 = getallcited2tpc(lda,texts,filename,Year)

logging.info("fig formation")

grap(tpc1,tpc2,num_topics,filename)

if __name__ == "__main__":

Year = 2022

main(Year)

According to the LDA result, you only need to adjust the k value in the code. K represents the total number of topics. The selection of the k value is based on multiple indicators, and you can select according to your results. The results will finally output two files, the calculation result and the visualization result, based on Gepia software, which can be displayed in various ways. The results can refer to Figure 5 and Figure 6 of the manuscripte.
